# Predictors of Infused Distending Fluid Volume in Hysteroscopic Myomectomy

**DOI:** 10.3390/medicina60091424

**Published:** 2024-08-30

**Authors:** Chia-Han Chung, Chien-Chen Tsai, Hsiao-Fen Wang, Hui-Hua Chen, Wan-Hua Ting, Sheng-Mou Hsiao

**Affiliations:** 1 Department of Obstetrics and Gynecology, Far Eastern Memorial Hospital, Banqiao District, New Taipei 220216, Taiwan; frank820130@gmail.com (C.-H.C.); christine06183@gmail.com (H.-F.W.); thandaaye24@gmail.com (H.-H.C.); stellatingwh@gmail.com (W.-H.T.); 2 Department of Anatomic Pathology, Far Eastern Memorial Hospital, Banqiao District, New Taipei 220216, Taiwan; a0970295250@gmail.com; 3 Department of Industrial Management, Asia Eastern University of Science and Technology, New Taipei 220303, Taiwan; 4 Department of Obstetrics and Gynecology, National Taiwan University College of Medicine and National Taiwan University Hospital, Taipei 100226, Taiwan; 5 Graduate School of Biotechnology and Bioengineering, Yuan Ze University, Taoyuan 320315, Taiwan

**Keywords:** hysteroscopy, leiomyoma, pulmonary edema, uterine myomectomy, water intoxication

## Abstract

*Background and Objectives*: The use of a bipolar resectoscope has become popular due to the lower risk of hyponatremia. However, gynecologists might overlook the risk of water intoxication. Water intoxication is associated with the infusion of distending fluid. We were interested in the prediction of the infused distending fluid volume in the era of bipolar hysteroscopy. Thus, the aim of this study was to identify the predictors of the infused distending fluid volume for hysteroscopic myomectomy. *Materials and Methods*: All consecutive women who underwent monopolar (n = 45) or bipolar (n = 137) hysteroscopic myomectomy were reviewed. *Results*: Myoma diameter (cm, coefficient = 680 mL, 95% confidence interval (CI) = 334–1025 mL, *p* <0.001) and bipolar hysteroscopy (coefficient = 1629 mL, 95% CI = 507–2752 mL, *p* = 0.005) were independent predictors of infused distending fluid volume. A myoma diameter ≥4.0 cm was the optimal cutoff value to predict the presence of >5000 mL of infused distending fluid. One woman in the bipolar group developed life-threatening water intoxication. *Conclusions*: Myoma diameter is associated with an increase in infused distending fluid volume, especially for myomas ≥4 cm. Meticulous monitoring of the infused distension fluid volume is still crucial to avoid fluid overload during bipolar hysteroscopic myomectomy.

## 1. Introduction 

Submucous myoma represents approximately 5–10% of all uterine myomas [1]. Submucous myoma is associated with abnormal uterine bleeding, infertility, and adverse pregnancy outcomes including multiple pregnancy losses [2].

Hysteroscopy is used to diagnose and treat a series of intrauterine disorders, including submucous myoma [2]. Carbon dioxide was first used to visualize the uterine cavity, and the use of liquid media has become prevalent since the 1980s [3]. Electrolyte-free solution is needed for hysteroscopy using a monopolar resectoscope, whereas isotonic electrolyte-rich medium (such as normal saline) is needed for hysteroscopy with a bipolar resectoscope. 

Complications of hysteroscopy include uterine perforation, fluid overload, hemorrhage, gas embolism, adjacent organ injury, hyponatremia, and lung edema. The bipolar resectoscope has become more popular than the monopolar resectoscope due to the lower risk of hyponatremia. Two randomized controlled trials did not show any differences in perioperative parameters between the monopolar and bipolar groups, except the risk of hyponatremia and hyposmolarity [4,5]. However, fluid overload might occur during bipolar hysteroscopic procedures, despite the lower risk of hyponatremia. Fluid overload could result in congestive heart failure and pulmonary edema. Additionally, Berg et al. found that fluid deficit was higher in the bipolar hysteroscopy group, compared to monopolar [6]. In our hospital, it seems that some gynecologists overlook the risk of fluid overload while performing bipolar hysteroscopic myomectomy. Thus, we were interested in whether there were any differences in the perioperative parameters (especially for infused distending fluid volume) between the monopolar and bipolar groups. The aim of this study was to identify perioperative parameters that can predict infused distending fluid volume in hysteroscopic myomectomy.

## 2. Materials and Methods

Between January 2009 and July 2021, the medical records of all consecutive women who underwent hysteroscopic myomectomy in a tertiary referral center were reviewed. Women with a prolapsed pedunculated submucous myoma, cervical myoma, or missing volumes of infused and collected fluids were excluded. Before January 2016, distilled water was used as the distending media for monopolar hysteroscopic myomectomy (i.e., the monopolar group) in our hospital. After February 2016 in our institution, a bipolar resectoscope was used and normal saline was used as the distending media (i.e., the bipolar group). The Research Ethics Review Committee of Far Eastern Memorial Hospital approved this study. The requirement for informed consent was waived due to the retrospective nature of the study.

Baseline characteristics and perioperative data, such as myoma diameter, myoma weight, volumes of infused and collected distending fluid, blood loss volume, operative time, and complications were recorded. Fluid deficit was calculated by subtracting the volume of the collected distending fluid from the volume of the infused distending fluid.

The hysteroscopic procedure was performed according to the following steps: Under intravenous or mask anesthesia, cervical dilatation was performed with a Hegar dilator. Some patients received a 10 mL injection of dilute vasopressin solution (0.2 U/mL) into the intracervical stroma at 3- and 9-o’clock positions [7]. Next, a monopolar resectoscope with an outer diameter of 8 mm (Karl Storz, Tuttlingen, Germany) or a bipolar resectoscope with an outer diameter of 8 mm (Olympus, Hamburg, Germany) was inserted into the uterine cavity. The uterine cavity was then infused with distilled water or normal saline for hysteroscopic myomectomy. 

A large collecting bag was tucked beneath the woman’s gluteal region and secured to the surgeon’s gown to capture fluid that spilled from the cervix and the resectoscope. The total volumes of infused fluid and outflow fluid were recorded. The methods for delivery of the infused intrauterine fluid included the manual infusion [8,9], pump infusion, or gravity methods. 

In the manual infusion method, a 60 mL disposable syringe (BD Plastipak TM, BD Medical, County Louth, Ireland) was connected to the resectoscope via a 90 cm extension tube (Sigma, Sigma Medical Supplies Corp., Taoyuan, Taiwan), and an assistant helped to pump the distilled water manually. Two 60 mL syringes were used at one time to minimize the waiting time required for refilling the syringe [9]. 

In the pump infusion method, a continuous-flow fluid infusion pump device (1 L Pressure Infuser Irrigation Pump, ConMed, Utica, NY, USA) was used to deliver fluid media with a compression cuff set at an initial pressure of 70 mmHg. Infusion pressure could be adjusted to 100 mmHg to reach adequate visibility [9]. 

In the gravity method, the infusion bag was set 100 to 150 cm above the patient’s level to create a hydrostatic pressure of approximately 70 to 100 mmHg at the level of the patient’s uterus.

Stata software (Version 11.0; Stata Corp, College Station, TX, USA) was used for statistical analyses. A *p* value of less than 0.05 was considered statistically significant. The Wilcoxon rank-sum test or chi-square test was performed as appropriate. Multivariable backward stepwise linear regression analysis was performed using all variables in the univariate analysis to predict the volume of the infused distending fluid until all remaining variables had *p* < 0.05. Receiver operating characteristic (ROC) curve analysis was performed to identify the optimal cutoff value for differentiating >5000 mL infused distending fluid. The optimal cutoff value was determined by the point on the ROC curve closest to the upper left-hand corner.

## 3. Results

**Figure 1:**
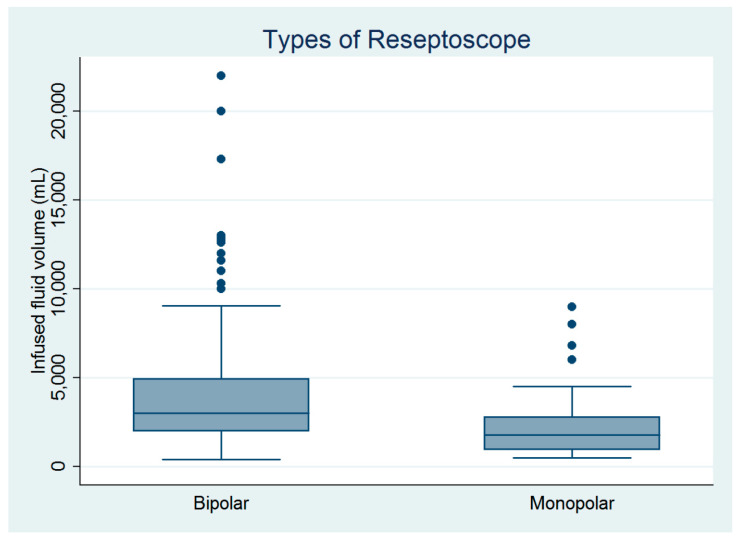
Comparison of the infused distending fluid volumes between the monopolar and bipolar groups (n = 182).

There were 182 women who underwent hysteroscopic myomectomy in this study (Table 1). Hysteroscopic myomectomy was performed by four gynecologic surgeons. Except for age, parity, and surgeons, the other baseline characteristics did not differ between the monopolar and bipolar groups (Table 1). However, the infused distending fluid volume was significantly larger in the bipolar group than in the monopolar group (Figure 1), despite no between-group differences in blood loss volume, operative time, or complication rate (Table 1). The estimated powers for a two-sample comparison of means of infused distending fluid volume, collected distended fluid volume, and fluid deficits were 0.995, 0.995, and 0.245, respectively (Table 1).

In addition, one woman in the bipolar group developed a life-threatening pulmonary edema after an infusion of 22,000 mL of normal saline as the distending fluid for treating a 6.5 cm type 0 submucous myoma. This patient was transferred to the intensive care unit after resuscitation, transferred to the general ward three days later, and discharged after a 14-day hospital stay. This patient did not have any long-term complications during follow-up.

**Figure 2:**
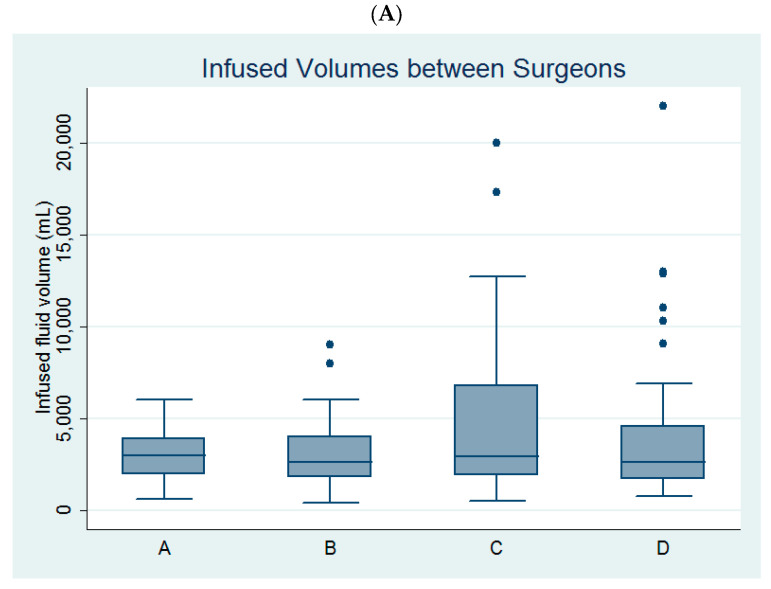

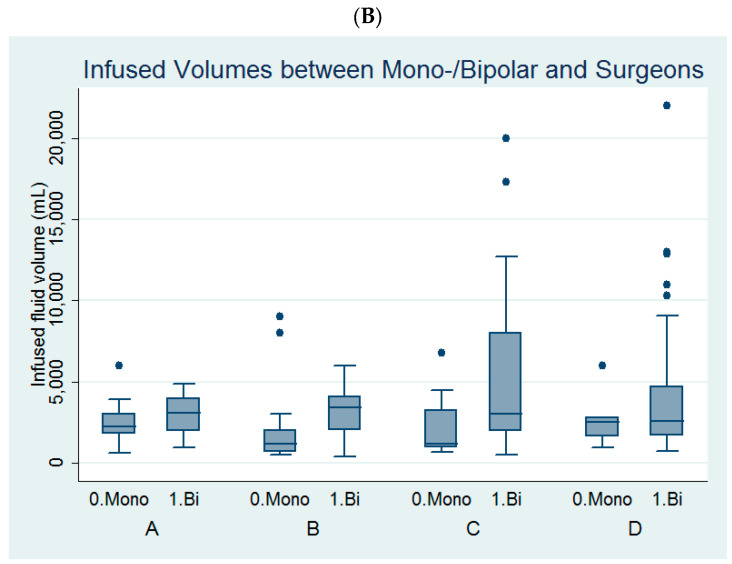
Comparison of (**A**) the infused distending fluid volume between the four surgeons and (**B**) the infused distending fluid volumes between the monopolar and bipolar groups among the four surgeons.

Univariate linear regression analysis revealed that age, parity, myoma diameter, the use of bipolar resectoscope, and surgeon C (Figure 2A) were predictors of infused distending fluid volume (Table 2).

Multivariable backward stepwise linear regression analysis revealed that myoma diameter (coefficient = 680 mL, 95% confidence interval (CI) = 334–1025 mL, *p* <0.001) and the use of bipolar hysteroscopy (coefficient = 1629 mL, 95% CI = 507–2752 mL, *p* = 0.005) were independent predictors of infused distending fluid volume with a constant of 1629 mL (95% CI = 507–2752 mL, *p* = 0.005). Thus, the predicted infused distending fluid volume (y) for a given myoma diameter (a) and the use of a bipolar resectoscope (b = 1; otherwise, b = 0) can be denoted by y =680x a + 1629x b +262. Therefore, an infused distending fluid volume of 4611 mL can predict the need for the treatment of a 4 cm submucous myoma using a bipolar resectoscope.

Among the four surgeons, surgeon B and surgeon C had larger volumes of infused distending fluid in the bipolar group than in the monopolar group (Table 3, Figure 2B).

**Figure 3:**
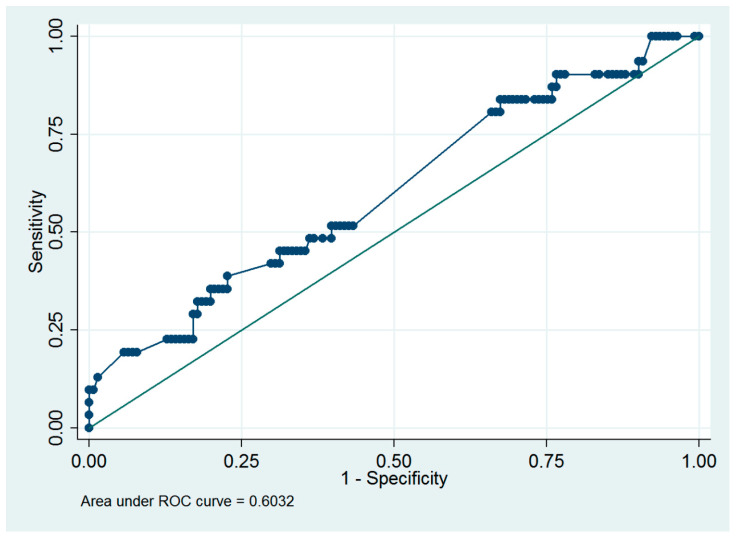
Receiver operating characteristic curve of using the myoma diameter to predict the presence of >5000 mL of the infused distending fluid in women who underwent hysteroscopic myomectomy.

Based on a previous study [8], an infused distending fluid volume of 5000 mL was suggested to be the cutoff value for the surgeon to consider early termination of a hysteroscopic procedure. Thus, ROC analysis was performed to predict the presence of >5000 mL of infused distending fluid volume, and myoma diameter ≥4.0 cm was the optimal cutoff value, with an ROC area of 0.60 (95% CI = 0.49 to 0.72; sensitivity = 38.7%, specificity = 77.3%, Figure 3).

A literature review was performed to search for cases involving the occurrence of pulmonary edema in women receiving hysteroscopic myomectomy. Including our case, a total of 9 cases including monopolar (n = 4) and bipolar (n = 5) hysteroscopic myomectomy have been reported [10,11,12,13,14,15,16] (Table 4).

## 4. Discussion

Ting et al. reported that 5000 mL of infused distending fluid was recommended as the cutoff value for the surgeon to consider termination of a hysteroscopic procedure to avoid a large fluid deficit [8]. Our study revealed that a myoma diameter ≥4.0 cm was the optimal cutoff value to predict the presence of >5000 mL of infused distending fluid. In other words, when treating a ≥4 cm submucous myoma, the surgeon should be cautious and closely monitor the volume of infused distending fluid and fluid deficit. Two-step hysteroscopic myomectomy should be considered in cases with ≥4.0 cm myoma. Similarly, Litta et al. reported that all intraoperative complications occurred when the myoma diameter was >3.75 cm [17]. It is worth mentioning that when limiting the infused distending fluid to 2000 mL, no serious complications associated with irrigation media are expected [18].

In our study, the bipolar resectoscope was an independent predictor of a larger infused distending fluid volume (coefficient = 1629 mL, *p* = 0.005, Table 2). That is, the use of a bipolar resectoscope was associated with an increase in infused distending fluid volume in our hospital. Bipolar hysteroscopy is safer than monopolar hysteroscopy due to the lower risk of hyponatremia. However, gynecologists may overlook the risk of fluid overload while performing bipolar hysteroscopic myomectomy. The above might explain our finding of an increase in infused distending fluid volume in the bipolar group.

An increase in infused distending fluid volume was associated with an increase in fluid deficit (Spearman rho = 0.24, *p* = 0.001) in our study. Berg et al. also reported that the fluid deficit was higher in the bipolar group than in the monopolar group (1227 mL versus 765 mL, *p* < 0.01) [6]. Their finding of an increased fluid deficit was in line with our finding of an increased infused distending fluid volume in the bipolar group.

Nonetheless, two randomized controlled trials did not show any differences in perioperative parameters between the monopolar and bipolar groups, except the risk of hyponatremia and hyposmolarity [4,5]. The real reason for the above discrepancy between our data and that in the above two randomized control trials might be related to the Hawthorne effect [19,20]. In a randomized clinical trial, it was impossible to mask the group assignments, and subconscious bias could have affected the threshold at which they performed hysteroscopic myomectomy [21]. The Hawthorne effect (i.e., the human tendency to improve performance because of the awareness of being studied [19,20]) might remind gynecologists about monitoring the infused distending fluid volume. Nonetheless, in the real world, gynecologists might not pay much attention to the infused distending fluid volume due to safer distending media (i.e., normal saline) being used in bipolar hysteroscopy.

Among the perioperative complications, fluid overload is the most common. Pulmonary edema, cerebral edema, and hyponatremia were commonly reported in patients who underwent monopolar hysteroscopy. These complications are infrequent but are associated with significant morbidity or mortality. Bipolar hysteroscopic myomectomy is safer than monopolar due to its lower risk of hyponatremia [22]. Nonetheless, four life-threatening fluid overload events have been reported in women who underwent bipolar hysteroscopic myomectomy [14,15,16] (Table 4). Therefore, the meticulous monitoring of infused distending fluid volume and fluid deficit is also imperative during bipolar hysteroscopic myomectomy.

A level of 50–60 mmHg has been suggested to be the optimal infusion pressure during outpatient diagnostic hysteroscopy [23,24]. Nonetheless, Karaman et al. suggested to start with 20 mmHg as the initial infusion pressure and increase the pressure gradually to higher levels, such as 50, 70, and 100 mmHg, to reach adequate visibility [25]. However, higher infusion pressure is associated with higher pain scores during hysteroscopy, and there was no difference in pain scores measured 30 min after hysteroscopy between the lower (30–50 mmHg) and higher infusion pressure (70–100 mmHg) groups [25]. Thus, for those women who receive anesthesia during hysteroscopic myomectomy, it seems reasonable to use 70 mmHg as the initial infusion pressure and increase the pressure to 100 mmHg to reach adequate visibility.

It is worth mentioning that the systemic absorption of infused distending fluid increases considerably when the intrauterine pressure exceeds the mean arterial pressure [26], and the lower mean arterial pressure, the lower the infusion pressure that should be used to minimize systemic absorption [27]. The mean arterial pressure in normal healthy people has been reported to be 88 ± 9 mmHg [28]. Hasham et al. also reported that there was no absorption of the distending fluid into the venous system at an intrauterine pressure of 70 mmHg, while contrast distending medium was clearly seen entering the uterine venous plexus at 150 mmHg [29]. Thus, the estimated infused pressure of 70–100 mmHg in our pump infusion and gravity methods seems to be reasonable.

We did not use an automatic fluid deficit control system in our hospital [30,31]. Thus, we could not monitor the difference between the infused and collected fluids simultaneously during hysteroscopic myomectomy. After the occurrence of pulmonary edema in the abovementioned woman, we proposed a protocol for monitoring the fluid deficit if the infused distending fluid volume was >4000 mL, similar to Alexandroni et al.’s report [32].

This study is limited due to its retrospective, nonrandomized nature and small sample size. Retrospective studies are subject to biases such as selection bias and recall bias, which can affect the reliability of the findings. Additionally, this research was conducted at a single tertiary referral center, which may limit the generalizability of the results. Furthermore, this study did not use an automatic fluid deficit control system, which could have provided more accurate and real-time monitoring of fluid balance. Manual monitoring is prone to human error, which can affect the accuracy of the fluid measurements. However, we presented real-world data about the switch from monopolar to bipolar hysteroscopic myomectomy. One case of life-threatening water intoxication was found in the bipolar group, and this should have clinical implications.

The study involved four different surgeons, and there was variability in the infused distending fluid volumes among them. This introduces another layer of variability that could influence the results. Standardizing the surgical technique and fluid management protocol across all surgeons could minimize this variability. Our hospital established a fluid management protocol after the life-threatening event. Future research should be conducted to assess the effect of this protocol.

Certain patients were excluded from the study, such as those with prolapsed pedunculated submucous myoma, cervical myoma, or missing volumes of infused and collected fluids. Most prolapsed pedunculated submucous myoma or cervical myoma can be easily treated by ligating and cutting the stalk of the myoma from the vagina. Thus, the mean infused fluid volume for treating these prolapsed and cervical myomas should be lower than that for intrauterine submucous myomas. Therefore, we excluded prolapsed pedunculated myoma and cervical myoma to minimize bias. However, these exclusions may limit the applicability of the findings to all patients undergoing hysteroscopic myomectomy.

## 5. Conclusions

In conclusion, myoma diameter is associated with an increase in infused distending fluid volume, especially for myomas ≥4 cm. Meticulous monitoring of infused distending fluid volume and fluid deficit is still crucial to avoid fluid overload during bipolar hysteroscopic myomectomy.

## Figures and Tables

**Table 1:** Comparisons of the baseline and perioperative data of women who underwent monopolar versus bipolar hysteroscopic myomectomy (n = 182).

Variables	Monopolar Group (n = 45)	Bipolar (n = 137)	*p* †	Power
Age (years)	48.9 ± 7.6	44.0 ± 8.3	0.0002	0.956
Body mass index (kg/m^2^)	20.1 ± 2.2	20.6 ± 3.2	0.54	0.216
Parity	2.10 ± 1.30	1.46 ± 1.17	0.04	0.835
Hemoglobin (g/dL)	8.8 ± 2.9	10.0 ± 2.1	0.09	0.727
Myoma diameter(cm)	3.1 ± 1.3	3.4 ± 1.4	0.22	0.261
Myoma weight (g)	23.0 ± 45.3	25.4 ± 28.4	0.11	0.063
Infused distending fluid (mL)	2317 ± 2024	4310 ± 3761	<0.0001	0.995
Collected distending fluid (mL)	2124 ± 1920	4000 ± 3469	<0.0001	0.995
Fluid deficit (mL)	193 ± 289	310 ± 957	0.77	0.245
Blood loss (mL)	58 ± 75	83 ± 125	0.12	0.366
Operative time (min)	33.7 ± 15.6	39.5 ± 19.8	0.18	0.523
Inpatient treatment	22 (49)	95 (69)	0.01	0.610
Length of stay (days)	1.7 ± 0.8	2.0 ± 1.1	0.06	0.311
Perioperative complication	0 (0)	1 (1)	0.57	0.002
Surgeon				
A	10 (22)	11 (8)	0.005	-
B	18 (40)	36 (26)		
C	12 (27)	55 (40)		
D	5 (11)	35 (26)		

Values are expressed as the mean ± standard deviation or number (percentage). † Wilcoxon rank-sum test or chi-square test.

**Table 2:** Factors predicting the infused distending fluid volume (mL, n = 182).

	Univariate		Multivariable	
Variables	Coefficient (mL)	*p* †	Coefficient (mL)	*p* ‡
Age (years)	−97 (−157, −37)	0.002	-	-
Body mass index (kg/m^2^)	−165 (−409, 78)	0.18	-	-
Parity	−928 (−1558, −298)	0.004	-	-
Hemoglobin (g/dL)	90 (−204, 386)	0.55	-	-
Myoma diameter (cm)	726 (374, 1077)	<0.001	680 (334, 1025)	<0.001
Operative time (min)	13.3 (−14.5, 41.1)	0.35	-	-
Bipolar resectoscope	1483 (381, 2585)	0.009	1629 (507, 2752)	0.005
Surgeon				
A (reference)	-	-	-	-
B	82 (−1673, 1837)	0.93	-	-
C	1760 (54, 3466)	0.04	-	-
D	1373 (−465, 3212)	0.14	-	-

Values are expressed as coefficients (95% confidence interval). † Linear regression analysis. ‡ Multivariable backward stepwise linear regression analysis were performed using all variables in the univariate analysis until all remaining variables had *p* <0.05. R^2^ = 0.13.

**Table 3:** Comparisons of infused distending fluid volumes (mL) between the monopolar and bipolar groups among the four surgeons.

Surgeon	Monopolar	Bipolar	*p*
A	2630 ± 1496	3036 ± 1204	0.31
B	2087 ± 2438	3344 ± 1372	0.0003
C	2213 ± 1926	5125 ± 4408	0.007
D	2770 ± 1949	4423 ± 4558	0.45

Values are expressed as the mean ± standard deviation.

**Table 4:** Occurrence of pulmonary edema in women who underwent hysteroscopic myomectomy.

Case No	Author	Monopolar or Bipolar	Infused Fluid	Infused Fluid Amount (mL)	Fluid Deficit (mL)	Complication
1	Jo et al. [10]	Monopolar	5:1 sorbitol/mannitol	2000	-	Hyponatremia, pulmonary edema
2	Woo et al. [11]	Monopolar	2.7% sorbitol/0.54% mannitol	24,000	-	Hyponatremia, pulmonary edema
3	Elegante et al. [12]	Monopolar	1.5% Glycine	-	2700	Hyponatremia, cerebral edema, pulmonary edema
4	Liao et al. [13]	Monopolar	5% dextrose water	8000	-	Cardiac arrest, pulmonary edema, hyponatemia
5	Grove et al. [14]	Bipolar	Lactated Ringer’s solution	18,000	6000	Pulmonary edema
6	Van Kruchten et al. [15]	Bipolar	Normal saline	-	-	Pulmonary edema
7	Wang et al. [16]	Bipolar	Normal saline	19,100	11,900	Pulmonary edema
8	Wang et al. [16]	Bipolar	Normal saline	7500	4500	Pulmonary edema
9	This study	Bipolar	Normal saline	22,000	-	Pulmonary edema

## Data Availability

Data are available on request from the corresponding author.

## References

[1] American Association of Gynecologic Laparoscopists (AAGL): Advancing Minimally Invasive Gynecology Worldwide (2012). AAGL practice report: Practice guidelines for the diagnosis and management of submucous leiomyomas. J. Minim. Invasive Gynecol..

[2] American College of Obstetricians and Gynecologists (2005). ACOG technology assessment in obstetrics and gynecology, number 4, August 2005: Hysteroscopy. Obstet. Gynecol..

[3] Marlow J.L. (1995). Media and delivery systems. Obstet. Gynecol. Clin. N. Am..

[4] Kansal Y., Roy K.K., Metta S., Kumar S., Singhal S., Vanamail P. (2017). A Prospective randomized study comparing unipolar versus bipolar hysteroscopic myomectomy in infertile women. J. Hum. Reprod. Sci..

[5] Darwish A.M., Hassan Z.Z., Attia A.M., Abdelraheem S.S., Ahmed Y.M. (2010). Biological effects of distension media in bipolar versus monopolar resectoscopic myomectomy: A randomized trial. J. Obstet. Gynaecol. Res..

[6] Berg A., Sandvik L., Langebrekke A., Istre O. (2009). A randomized trial comparing monopolar electrodes using glycine 1.5% with two different types of bipolar electrodes (TCRis, Versapoint) using saline, in hysteroscopic surgery. Fertil. Steril..

[7] Phillips D.R., Nathanson H.G., Milim S.J., Haselkorn J.S. (1997). The effect of dilute vasopress in solution on the force needed for cervical dilatation: A randomized controlled trial. Obstet. Gynecol..

[8] Ting W.-H., Lin H.-H., Wu M.-P., Tu F.-C., Peng F.-S., Hsiao S.-M. (2015). Safety and efficacy of manual syringe infusion of distending media for hysteroscopic procedures: A case-control study. Eur. J. Obstet. Gynecol. Reprod. Biol..

[9] Ting W.H., Lin H.H., Hsiao S.M. (2019). Manual versus pump infusion of distending media for hysteroscopic procedures: A randomized controlled trial. Sci. Rep..

[10] Jo Y.Y., Jeon H.J., Choi E., Choi Y.S. (2011). Extreme hyponatremia with moderate metabolic acidosis during hysteroscopic myomectomy—A case report. Korean J. Anesthesiol..

[11] Woo Y.C., Kang H., Cha S.M., Jung Y.H., Kim J.Y., Koo G.H., Park S.G., Baek C.W. (2011). Severe intraoperative hyponatremia associated with the absorption of irrigation fluid during hysteroscopic myomectomy: A case report. J. Clin. Anesth..

[12] Elegante M., Hamera J., Xiao J., Berger D. (2019). Operative hysteroscopy intravascular absorption syndrome causing hyponatremia with associated cerebral and pulmonary edema. Clin. Pract. Cases Emerg. Med..

[13] Liao C.-Y., Lo C.-H., Yu M.-X., Chan W.-H., Wei K.-Y., Tseng M.-F., Wu C.-C. (2020). Life-threatening acute water intoxication in a woman undergoing hysteroscopic myomectomy: A case report and review of the literature. BMC Womens Health.

[14] Grove J.J., Shinaman R.C., Drover D.R. (2004). Noncardiogenic pulmonary edema and venous air embolus as complications of operative hysteroscopy. J. Clin. Anesth..

[15] Van Kruchten P.M., Vermelis J.M., Herold I., van Zundert A.A. (2010). Hypotonic and isotonic fluid overload as a complication of hysteroscopic procedures: Two case reports. Minerva Anestesiol..

[16] Wang M.T., Chang C.C., Hsieh M.H., Chang C.W., Chiang YH F., Tsai H.C. (2020). Operative hysteroscopy intravascular absorption syndrome is more than just the gynecological transurethral resection of the prostate syndrome: A case series and literature review. Taiwan J. Obstet. Gynecol..

[17] Litta P., Leggieri C., Conte L., Dalla Toffola A., Multinu F., Angioni S. (2014). Monopolar versus bipolar device: Safety, feasibility, limits and perioperative complications in performing hysteroscopic myomectomy. Clin. Exp. Obstet. Gynecol..

[18] Tammam A.E., AHmed H.H., Abdella A.H., Taha S.A.M. (2015). Comparative study between monopolar electrodes and bipolar electrodes in hysteroscopic surgery. J. Clin. Diagn. Res..

[19] Roethlisberger F.J., Dickson W.J. (1939). Management and the Worker.

[20] Wickstrom G., Bendix T. (2000). The ‘Hawthorne effect’—What did the original Hawthorne studies actually show?. Scand. J. Work Environ. Health.

[21] Davey M.A. (2018). Labor induction vs. Expectant management of low-risk pregnancy. N. Engl. J. Med..

[22] Colacurci N., de Franciscis P., Mollo A., Litta P., Perino A., Cobellis L., de Placido G. (2007). Small-diameter hysteroscopy with Versapoint versus resectoscopy with a unipolar knife for the treatment of septate uterus: A prospective randomized study. J. Minim. Invasive Gynecol..

[23] Haggag H.M., Hassan A.M. (2016). The impact of altering filling pressures in diagnostic outpatient hysteroscopy on the procedure completion rates and associated pain: A randomized double blind controlled trial. Aust. N. Z. J. Obstet. Gynaecol..

[24] Haggag H., Hassan A., Wahba A., Joukhadar R. (2017). A randomized double-blind controlled trial of different filling pressures in operative outpatient hysteroscopy. Int. J. Gynaecol. Obstet..

[25] Karaman E., Kolusarı A., Çetin O., Çim N., Alkış I., Karaman Y., Güler S. (2017). What should the optimal intrauterine pressure be during outpatient diagnostic hysteroscopy? A randomized comparative study. J. Obstet. Gynaecol. Res..

[26] Garry R., Hasham F., Kokri M.S., Mooney P. (1992). The effect of pressure on fluid absorption during endometrial ablation. J. Gynecol. Surg..

[27] Umranikar S., Clark T.J., Saridogan E., Miligkos D., Arambage K., Torbe E., Campo R., Sardo A.D.S., Tanos V., Grimbizis G. (2016). BSGE/ESGE guideline on management of fluid distension media in operative hysteroscopy. Gynecol. Surg..

[28] Herbert A., Cruickshank J.K., Laurent S., Boutouyrie P. (2014). Establishing reference values for central blood pressure and its amplification in a general healthy population and according to cardiovascular risk factors. Eur. Heart J..

[29] Hasham F., Garry R., Kokri M.S., Mooney P. (1992). Fluid absorption during laser ablation of the endometrium in the treatment of menorrhagia. Br. J.Anaesth..

[30] Tomazevic T., Savnik L., Dintinjana M., Ribic-Pucelj M., Pompe-Tansek M., Vogler A., Kos D. (1998). Safe and effective fluid management by automated gravitation during hysteroscopy. J. Soc. Laparoendosc. Surg..

[31] Bahar R., Shimonovitz M., Benshushan A., Shushan A. (2013). Case-control study of complications associated with bipolar and monopolar hysteroscopic operations. J. Minim. Invasive Gynecol..

[32] Alexandroni H., Bahar R., Chill H.H., Karavani G., Ben-Yossef O., Shushan A. (2017). Reducing fluid-related complications during operative hysteroscopy: Use of a new mandatory fluid-balance form. J. Minim. Invasive Gynecol..

